# Advancing early warning capabilities with CHIRPS-compatible NCEP GEFS precipitation forecasts

**DOI:** 10.1038/s41597-022-01468-2

**Published:** 2022-06-30

**Authors:** Laura Harrison, Martin Landsfeld, Greg Husak, Frank Davenport, Shraddhanand Shukla, William Turner, Pete Peterson, Chris Funk

**Affiliations:** University of California Santa Barbara, Climate Hazards Center and Department of Geography, Santa Barbara, USA

**Keywords:** Climate sciences, Water resources

## Abstract

CHIRPS-GEFS is an operational data set that provides daily bias-corrected forecasts for next 1-day to ~15-day precipitation totals and anomalies at a quasi-global 50-deg N to 50-deg S extent and 0.05-degree resolution. These are based on National Centers for Environmental Prediction (NCEP) Global Ensemble Forecast System version 12 (GEFS v12) precipitation forecasts. CHIRPS-GEFS forecasts are compatible with Climate Hazards center InfraRed Precipitation with Stations (CHIRPS) data, which is actively used for drought monitoring, early warning, and near real-time impact assessments. A rank-based quantile matching procedure is used to transform GEFS v12 “reforecast” and “real-time” forecast ensemble means to CHIRPS spatial-temporal characteristics. Matching distributions to CHIRPS makes forecasts better reflect local climatology at finer spatial resolution and reduces moderate-to-large forecast errors. As shown in this study, having a CHIRPS-compatible version of the latest generation of NCEP GEFS forecasts enables rapid assessment of current forecasts and local historical context. CHIRPS-GEFS effectively bridges the gap between observations and weather predictions, increasing the value of both by connecting monitoring resources (CHIRPS) with interoperable forecasts.

## Abstract (summary)

**Table:** 

Measurement(s)	volume of hydrological precipitation
Technology Type(s)	forecast bias correction
Sample Characteristic - Organism	precipitation
Sample Characteristic - Environment	climate system
Sample Characteristic - Location	global

## Background & Summary

This article describes a precipitation forecast data set designed to support drought early warning and anticipate weather impacts across many regions of the globe. This data set bridges a gap between a resource that is actively used for monitoring agro-climatic conditions and the forward-looking information that modern numerical weather prediction (NWP) systems provide. The hope in creating this product is that people engaged in weather and climate-impact assessments, particularly those doing so to assist vulnerable communities, can more easily take advantage of the value of NWP data for new scientific applications, and for timely assessments and communication about high-risk situations. NWP data is a crucial resource for short-term disaster planning, and accessibility of weather forecasts has led to uptake in an ever-increasing number of applications.

Numerical weather prediction systems use a powerful computing framework to model key atmospheric processes. To help set the initial atmospheric state and to provide up-to-date information about how the current atmospheric state is evolving, satellite data and weather observations are assimilated into the model simulations several times a day. Weather prediction centers usually run a NWP model multiple times with perturbed initial conditions to characterize uncertainty. A major challenge to weather prediction, and a fundamental limit to predicting weather patterns and precipitation, is the so-called “butterfly effect,” named for the chaotic fluctuations in the atmosphere that amplify small anomalies over time^1^. Inaccurate representation of the current state and absent or poor parametrization of complex atmospheric processes in the NWP system also yield inaccuracies. Statistical post processing of NWP data is commonly done to correct for systematic errors that arise from resolution limitations and errors in the mean state and ensemble spread^2^. While numerical weather prediction is imperfect, users’ tolerance for error is situation-dependent, and, in some cases, a moderately skillful forecast can be invaluable.

In this data descriptor article, we present CHIRPS-GEFS, a precipitation forecast data product based on predictions from the widely used National Oceanic and Atmospheric Administration (NOAA) National Centers for Environmental Prediction (NCEP) Global Ensemble Forecast System (GEFS). CHIRPS-GEFS is an operational data set that uses quantile matching to increase the spatial resolution, remove systematic bias, and adjust the variance of deterministic precipitation forecasts from the newest version of this state-of-the-art numerical weather prediction system, GEFS version 12 (v12)^3^. CHIRPS-GEFS uses the Climate Hazards InfraRed Precipitation with Stations precipitation data product^4^ (CHIRPS) and GEFS v12 2000 to 2019 reforecast data^5^ for spatial downscaling and bias correction of 0.25-degree to 0.5-degree resolution GEFS v12 real-time ensemble mean forecasts. CHIRPS is a gridded merged satellite-station precipitation data product with a 40+ year record, quasi-global extent, and ~5 km resolution. CHIRPS-GEFS provides daily-updated 0.05-degree resolution forecasts for 1-day, 5-day, 10-day, and 15-day precipitation totals, as well as for pentads—the primary periodicity for CHIRPS data. CHIRPS and CHIRPS-GEFS data are produced by the University of California Santa Barbara (UCSB) Climate Hazards Center (CHC).

CHIRPS-GEFS is designed to support operational seasonal precipitation monitoring and impact forecasting by providing a version of GEFS forecasts that are compatible with CHIRPS data. CHIRPS is widely used for drought early warning, agro-climatological monitoring, and historical climate impact and trend assessments^6,7^. The goal is to produce forecasts and estimates with similar distributions, specifically, by adjusting the GEFS forecast mean and variance structure to be similar to CHIRPS. Matching distributions to accurate gridded precipitation estimates constrains forecasts to better reflect the local climatology, and it is a necessary preprocessing step for comparing forecasts to observed precipitation amounts. While it does not resolve inaccuracy in numerical weather predictions, the quantile matching process used to produce CHIRPS-GEFS removes systematic bias errors and can substantially improve the representation of precipitation in areas associated with complex terrain. Bias-corrected precipitation forecasts are important source of error reduction in hydrologic forecast applications^8,9^.

CHIRPS-GEFS is available as a downloadable data product, through data viewers, and through regional precipitation monitoring maps. In this article, we describe CHIRPS-GEFS forecast products and access. We show areas of the globe where the temporally varying bias correction produces large and small adjustments, and where forecasts for 5-day and 15-day precipitation totals tend to perform well or poorly, according to deterministic and categorical skill metrics. We also show an exciting operational application that seamlessly combines CHIRPS data and CHIRPS-GEFS forecasts to provide outlooks that support drought early warning in food-insecure countries.

## Methods

### Data sources: Climate Hazards center InfraRed Precipitation with Stations data and NCEP Global Ensemble Forecast System forecasts

Key strengths that make CHIRPS reliable for operational monitoring, and a good candidate for forecast bias correction, are its long record for historical context, low latency to support operations, low bias, and good performance in validation studies^4,10–27^. It combines satellite thermal infrared cold cloud duration-based precipitation estimates with *in situ* observations from a large and quality controlled archive of weather station reports from global, regional, and national meteorological networks. CHIRPS incorporates a relatively dense gauge network in typically underrepresented places, such as Ethiopia, Somalia, Southern Africa, Mexico, and Central America, as well as in the United States, Western Europe, South Africa, and Australia. CHIRPS has a low 2-day latency for preliminary pentads (~5 day periods) and a ~2.5 week latency for the final version of data for the previous month. CHIRPS has low bias^4^ compared to the high-quality gridded gauge-based Global Precipitation Climatology Centre data, which has a much longer year-plus latency. Low bias is primarily achieved by the CHIRPS algorithm for anomaly estimation being centered upon the high-quality ~5 km-resolution Climate Hazards Center’s Precipitation Climatology^28^ (CHP_clim_). CHP_clim_ uses moving window local regressions to predict monthly station climate normals using geographic attributes and long-term means from satellite precipitation estimates. Estimates are then refined in a two-step process using spatially interpolated (inverse distance weighted) model residuals and Global Historical Climatology Network version 2^29^ observed monthly means.

GEFS is an advanced NWP system that has been operated by the National Centers for Environmental Prediction since December 1992^30^. The latest version, GEFS version 12^3^, was implemented in September 2020 for operational forecasting and is the first global-scale coupled forecast system at NCEP following the United Forecast System (UFS) framework. The system produces operational “real-time” forecasts 4 times daily out to 16+ days. Significant system advances in GEFS v12 include the Global Forecast System model (GFSv15.1) with a computationally efficient Finite-Volume Cubed Sphere (FV3) dynamical core, down to 0.25-degree resolution and extended-length forecast outputs, more ensemble members, and improved model perturbation techniques, physical parameterization schemes, and global wave forecasts^31,32^. According to the NCEP Environmental Modeling Center (EMC) Model Evaluation Group (MEG), strengths of GEFS v12, compared to previous systems, include more reliable precipitation forecasts, improved representation of weather events near topography, increased ensemble spread, and improved synoptic-scale weather prediction. Relative to the older version, GEFS v12 has 25–30% higher Brier Skill Scores for day 1-to-5 quantitative precipitation forecasts in the continental United States, smaller errors in tropical cyclone tracks and intensity, and better prediction of the Madden-Julian Oscillation and other large-scale indices relevant to precipitation in the tropics and subtropics^32^. The MEG evaluation can be accessed at https://www.emc.ncep.noaa.gov/users/meg/gefsv12/. More on GEFS v12 can be found in Zhao *et al*.^3^ and on the NCEP webpage https://www.emc.ncep.noaa.gov/emc/pages/numerical_forecast_systems/gefs.php.

To make real-time GEFS forecasts compatible with CHIRPS data, CHIRPS-GEFS bias correction uses 2000 to 2019 GEFS v12 FV3 Phase 2 “reforecast” data described in Guan *et al*.^5^ and produced by NCEP EMC to accompany the GEFS v12 real-time forecasts. These data are managed by NOAA and can be accessed through the Registry of Open Data on Amazon Web Services (AWS, https://registry.opendata.aws/noaa-gefs-reforecast/). Reforecast data are retrospective weather forecasts that are generated from a fixed NWP model framework. They are a valuable resource for statistical correction of weather forecasts, as well as for model developers to diagnose model biases^2,33^. The reforecast data consists of daily GEFS forecasts, out to 16 days, for the period 2000 to 2019, for 5 ensemble members. The reforecast data were downloaded from https://noaa-gefs-retrospective.s3.amazonaws.com/index.html. Spatial resolution varies in the reforecast data, from 0.25 degrees for the first 10 days to 0.5 degrees after that. The reforecast project also includes a once-per-week, 11-member forecast to 35+ days. For CHIRPS-GEFS, the reforecast data and previous real-time data are used to place the current day’s 0000 UTC GEFSv12 real-time forecast in historical context. CHIRPS-GEFS uses the ensemble mean from both the reforecast data and the real-time forecasts.

An integral part of the implementation of the GEFS at NOAA was the simultaneous production of two reanalysis data sets and this companion reforecast data set. In post processing raw forecasts, it is ideal for the training data to have similar bias and error characteristics, so efforts were made by the NCEP EMC and the NOAA Earth System Research Laboratories (ESRL)/Physical Sciences Laboratory (PSL) to use modeling system configurations similar to the operational GEFS v12. However, these are not identical. Some characteristics are described here and details are provided by Guan *et al*.^5^ and Hamill *et al*.^2^. The Phase 2 reforecast data used initial conditions from the ESRL/PSL 20-year reanalysis and were generated from FV3 GFS/ensemble Kalman filter (EnKF) hybrid analyses and EnKF 6-hour forecasts with the incremental analysis update (IAU) replay process to improve accuracy and reduce noise^5^. The reanalysis’ control and perturbed members were run at coarser resolution than the real-time forecasts due to limited computational resources^2^. Among other differences are the boundary conditions, with the reforecast using Optimum Interpolation Sea Surface Temperature data instead of a more sophisticated two-tiered SST procedure^5^. Data-availability differences between reforecast and real-time data include fewer daily ensembles members (5 versus 31) and fewer initialization cycles per day. For a sense of the differences in bias that the non-identical configurations may produce, Hamill *et al*.^2^ compared reforecast data to a 2-year-long set of separate retrospective forecasts that closely mimic operational real-time GEFS v12 data production. They found similar errors in near-surface weather variables in both the reforecast ensemble mean and the retrospective ensemble mean, based on its first five ensemble members, and reported that even though there would be some differences, reforecast data should provide acceptable similarity for post processing. For impacts of these differences on CHIRPS-GEFS bias correction, deeper investigation into statistical qualities of the reforecast versus real-time ensembles would be worthwhile in the future as more data becomes available.

### CHIRPS-GEFS bias correction: Rank-based quantile matching

Bias correction is a post-processing step that involves comparing the forecast to historical model forecasts and historical observations, ideally using a historical record that provides a representative sample of the possible distribution of precipitation outcomes. While the overall aim is to improve forecast skill, the choice of correction strategies depends on the issue being targeted, and these, in turn, depend on user needs. For example, experiments in improving probabilistic precipitation forecasts have shown success using training data and historical forecast analogs^34^. Researchers have used bias-corrected weather to seasonal forecasts to drive land surface models to better anticipate flood events^35,36^, streamflow^37,38^, root-zone soil moisture deficits^39^, and potential drought^40^.

The method currently used in CHIRPS-GEFS production is a type of quantile matching, a common method of systematic bias correction and data downscaling that performs well for modeled hydroclimatic data^5,9,41–44^. The National Weather Service, for example, uses quantile matching to remove bias and calibrate 6-hourly forecasts in their U.S. National Blend of Models using multi-location past-60-day observations^45^. In quantile matching, forecast data are first translated from precipitation amounts into percentiles, based on historical forecast cumulative distribution functions, and then these are mapped to equivalent quantiles based on each location’s historical target distribution. In other words, if the original forecast amount was ranked high or low compared to past forecasts from 2000 to present, the bias-corrected forecast amount is equivalently ranked high or low compared to the observational record for the same period. This process adds local spatial information, increasing the effective resolution of the forecasts, and also produces forecasts that have statistical distributions that are similar to the observational data set. Hence, the resulting forecasts are both higher resolution and interoperable with CHIRPS observations.

Operational CHIRPS-GEFS data are a bias-corrected 0.05-degree version of the 0.25-degree resolution Day 1 to 10 and 0.5-degree resolution Day 11 to Day 16 GEFS real-time 00 UTC ensemble mean forecasts for total precipitation from https://ftp.ncep.noaa.gov/data/nccf/com/gens/prod/. This spatial-temporal resolution is comparable to the reforecast data, as described above.

On a daily basis, ensemble mean 6-hour forecast amounts are downloaded and summed for each of the 16 days. Day 11–16 data are resampled to 0.25-degree resolution with bilinear interpolation, and the daily totals are accumulated to the 16-day period. The rank-based quantile is calculated using sorted historical reforecast values and previous real-time forecasts for the same 16-day period for each pixel. Historical daily CHIRPS values are accumulated for the same time period and quantile-ranked for the same historical period. The corresponding CHIRPS rainfall amount is backed-out from the percentiles and used as the new CHIRPS-GEFS estimate. This 16-day estimate is then disaggregated to daily values using the original GEFS daily fractions of the 16-day prediction. These daily CHIRPS-GEFS values are accumulated to the 5-day, 10-day, 15-day CHIRPS-GEFS products every day. On the first day of each pentad and dekad, accumulations for those periods and the following two pentads are totaled as well.

Downscaling the GEFS 0.5-degree Day 11–16 forecasts to 0.25-degree resolution with bilinear interpolation introduces some precipitation changes, including smoother edges along storm precipitation gradients. Bilinear interpolation is not a mass-conserving approach. This influence was deemed acceptable for the purposes of this data product and upon consideration of the fact that storm details are uncertain at 11 to 16-day lead-times. A benefit of the smoothing at that step is that 0.5-degree edge discontinuities from those long-lead forecasts are not retained in the final data. The quantile matching makes use of the GEFS 16-day period percentiles at 0.25-degree; these are simply regridded to match the CHIRPS 0.05-degree resolution for that step.

Reforecast data is also downscaled and bias-adjusted using the same procedure as with the real-time data. This historical archive was used for skill assessments presented in this article.

## Data Records

CHIRPS-GEFS is available for public use as a downloadable data product, through interactive data viewers, and as regional monitoring maps. Operational and historical CHIRPS-GEFS data, based on bias-corrected GEFS v12 real-time data, from October 2020 to present, and Phase 2 reforecast data^46^ from 2000 to 2019, are located on a public data server. These data can be found at the UCSB CHC CHIRPS-GEFS webpage, where the CHIRPS-GEFS Precipitation Forecasts data repository^47^ (10.15780/G2PH2M) is also located. Users can cite the data as: “CHIRPS-GEFS Precipitation Forecasts. *CHIRPS-GEFS Data Repository* 10.15780/G2PH2M (2021). Data was accessed on [DATE].” The data are for use in operational precipitation monitoring and forecasting applications, for assessments like the current study on forecast performance, and for research and development. Data are in GeoTiff format, and are available for multiple variables and periodicities. Table 1 shows the main access points for CHIRPS-GEFS data and two online data viewers that include operational CHIRPS-GEFS data. Users can view images of the latest forecast 5-day, 10-day, and 15-day totals and anomalies at the CHIRPS-GEFS webpage, and can view and download time series (CSV format) and explore more images at the UCSB CHC and USGS Early Warning eXplorer (EWX) viewers.

**Table 1:** CHIRPS-GEFS Precipitation Forecasts: Data Access and Selected Data Viewers.

Resources	Description	Specifications	Access
**CHIRPS-GEFS precipitation forecast data**	Forecast 1-day, 5-day, 10-day, 15-day, pentad, and dekad precipitation totals for January 2000 to December 2019 and October 2020 to present.	50-deg S to 50-deg N at 0.05-deg resolution. Format: GeoTiff.	UCSB Climate Hazards Center. The CHIRPS-GEFS Precipitation Forecasts data repository^46^ contains all data and current forecast images 10.15780/G2PH2M.
**CHC Early Estimates**	Maps for monitoring conditions for past ~5 days to 3 months and region-specific seasons (CHIRPS final and preliminary data). A “+ Forecast’’ version shows a ~15 day outlook (CHIRPS-GEFS).	Precipitation totals, % average, historical rank, and more, for multiple regions. Format: PNG and GeoTiff.	UCSB Climate Hazards Center Monitoring and Forecasting webpage https://chc.ucsb.edu/monitoring
**Early Warning Explorer (EWX) Next Generation Viewers**	Graphical interfaces featuring multiple data sets including historical CHIRPS-GEFS (UCSB CHC EWX) and time series combining CHIRPS and CHIRPS-GEFS pentads (USGS EWX).	Time series for administrative and crop regions; images for user-defined extent. Format: GeoTiff, PNG and CSV.	UCSB CHC EWX https://chc.ucsb.edu/tools/ewx USGS EWX https://earlywarning.usgs.gov/fews/software-tools/1

Users can access CHIRPS-GEFS and CHIRPS data, and data that combines these, from the UCSB Climate Hazards Center. Table 1 describes several graphical applications that include CHIRPS-GEFS. Additionally, the CHIRPS-GEFS repository contains precipitation anomalies and standardized precipitation index (SPI) data for 5-day, 10-day, 15-day, pentad, and dekad periods (see Table 2), from October 2020.

Table 2 shows the update schedule for operational CHIRPS-GEFS data. The CHIRPS-GEFS data product includes daily-updated gridded 1-day, 5-day, 10-day, and 15-day daily forecast precipitation totals (mm), anomalies (mm), and standardized anomalies (z-scores), for quasi-global 50-deg N to 50-deg S extent at 0.05-degree resolution. Daily CHIRPS-GEFS updates are typically available shortly after the release of the GEFS forecast. Anomalies and z-scores are computed based on the entire reforecast and operational data record. File naming convention for 5-day, 10-day, and 15-day CHIRPS-GEFS indicates the day of GEFS forecast release and first day of the time period, which are the same. For example, the CHIRPS-GEFS 5-day forecast on January 1^st^ is the forecast precipitation total for the 1^st^ to the 5^th^.

**Table 2:** CHIRPS-GEFS Precipitation Forecasts: Data Update Schedule.

Forecast period	Update schedule	Variable
**1-day, 5-day, 10-day, 15-day**	Daily	Total
**5-day, 10-day, 15-day**	Daily	Anomaly and SPI
**Dekad** “First” forecast	16 days before the dekad ends	Total, Anomaly, and SPI
**Dekad** “Last” forecast	1st, 11th, 21st day of the month	Total, Anomaly, and SPI
**Pentad** “First,” “Second,” and “Last” forecasts	1st, 6th, 11th, 16th, 21st, 26th day of the month	Total

Forecast precipitation totals are provided for multiple periods to support applications. Precipitation totals and anomalies are in mm; standardized precipitation index (SPI) values are in number of standard deviations from the mean (z score). Anomalies and SPI are, at present, computed from all available years.

A second type of data update is also provided to support standard CHIRPS data temporal frequency and accumulations. On the 1^st^, 6^th^, 11^th^, 16^th^, 21^st^, and 26^th^ day of each month, daily forecast amounts from that day’s run are aggregated to pentads, the primary computing and update time step for CHIRPS v2 preliminary data. Pentads are the ~5-day periods that begin on these days of the month (e.g., the 6th pentad has 3 to 6 days, depending on the month). Similarly, on the 1^st^, 11^th^, and 21^st^, daily forecast amounts are aggregated to dekads to support applications like the GeoSpatial Water Requirement Satisfaction Index (GeoWRSI) that use dekadal data. Dekads are the ~10-day periods that begin on the 1^st^, 11^th^, and 21^st^. The pentad and dekad forecast amounts are named according to the order of update, with “First” designating the first opportunity to create the forecast using GEFS v12 forecasts (i.e., 16 days before the last day of the dekad), “Second” designating the second opportunity (for pentads only), and “Last” designating the last opportunity (i.e., on Day 1 of the period). GeoTiffs for pentad and dekad anomalies and totals are available from October 2020.

## Technical Validation

### Adjusting GEFS forecasts to local climatology

**Fig. 1:**
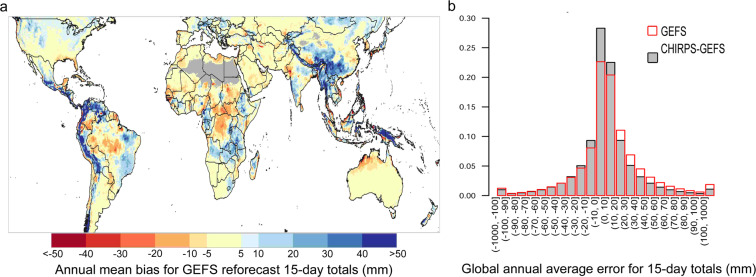
Annual mean bias and global error characteristics for GEFS reforecast data compared to CHIRPS, based on 15-day precipitation totals from Day 1, 6, 11, and 16 of each month during 2000–2019. Annual mean bias (**a**) shows the annual average of differences in GEFS reforecast and CHIRPS monthly means. Annual average error (**b**) shows the distribution of GEFS reforecast and CHIRPS-GEFS errors (product - CHIRPS). Both panels are based on in-season pixels, which are defined by monthly average CHIRPS > 10 mm.

What amount of correction is required for GEFS forecasts to align with CHIRPS local climatology? The amount of correction varies widely across the globe and throughout the year. Figure 1a shows annual mean bias for GEFS reforecast 15-day totals. In this figure, wetter-than-CHIRPS climatology and systematic over-prediction of 15-day totals by GEFS is indicated by positive mean bias values, while the opposite is indicated by negative values. GEFS forecast mean bias was calculated for each month and then averaged across rainy season months, to focus aggregate results on the rainfall seasons, when precipitation forecasts are relevant. Monthly dry masks excluded locations with a monthly average of less than 10 mm, according to CHIRPS climatology. In general, one consistent result from Fig. 1a is a tendency to increase precipitation in many mountainous tropical and subtropical regions. By design, orographic precipitation enhancements in such regions are represented fairly well in CHIRPS, and these are carried through to CHIRPS-GEFS precipitation forecasts. The CHIRPS-GEFS bias-correction process reduces systematic errors (Fig. 1b), with the overall mean absolute bias error going from 24.1 mm for GEFS to 19.7 mm for CHIRPS-GEFS, an ~18% reduction.

Figure 1a through Fig. 5 are based on GEFS reforecast, CHIRPS, and CHIRPS-GEFS data for the 5-day or 15-day periods beginning on the 1^st^, 6^th^, 11^th^, and 16^th^ day of the month. All these exclude dry season months. Figure 1b shows the corresponding global distribution of annual average error for the GEFS reforecast and CHIRPS-GEFS, and is discussed later.

GEFS has a large annual average positive bias of higher-than 40 mm in some areas of the globe, including in central Mexico, Central America, northern South America, the Andes and Himalayan Mountain ranges, and in southern China, Papua New Guinea, and localized areas of central Africa, the Ethiopian Highlands, and the western montane United States (Fig. 1a). GEFS has positive bias, by more than 5 mm for the annual average 15-day period, across the northern United States including in the Midwest, from Mexico’s northern mountains through most of Central America, in northern South America, the Andes range, eastern Brazil, in parts of central Europe, central and northern Asia, in the area from southern China to Myanmar and Thailand, and in northeastern and western India. GEFS has positive bias in portions of East Africa (Rwanda, Burundi, Tanzania, western Ethiopia), West Africa (Cameroon, Gabon), and Southern Africa (Zambia, central Angola, northern Zimbabwe, eastern South Africa). GEFS has negative bias, by more than 5 mm on average, in parts of central and northern Africa, Senegal, northern Australia, central South America, western India, the Yucatan peninsula, and the United States Gulf Coast.

**Fig. 2:**
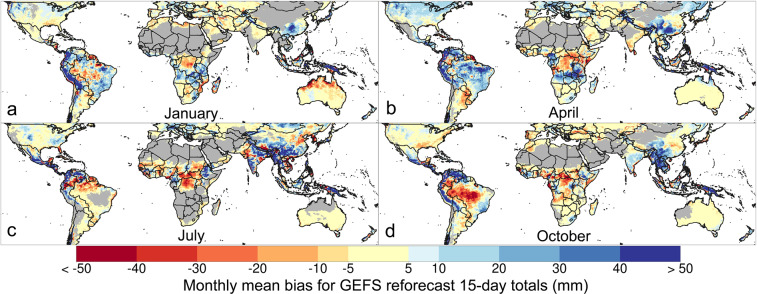
Monthly mean bias for GEFS reforecast data compared to CHIRPS, based on 15-day precipitation totals from Day 1, 6, 11, and 16 of each month during 2000–2019. Mean bias for January (**a**), April (**b**), July (**c**), and October (**d**) shows the difference in GEFS reforecast and CHIRPS monthly means. Shown for in-season pixels, which are defined by monthly average CHIRPS > 10 mm.

GEFS’ systematic bias changes throughout the year, as shown by the monthly mean bias in January, April, July, and October (Fig. 2). This is unsurprising, given that drivers of weather change too, but higher bias in particular months can be problematic for forecast users. In Ethiopia, for example, GEFS overestimates by large amounts during the Kirempt season (e.g., in July) and in October in the southwest. In central Brazil, the bias changes markedly by season, from a high negative bias in October to an expansive wet bias in April. In the Midwestern and northern United States, GEFS also shows a more expansive wet bias in April than in January, July, or October. In some areas, like in southern China and the Andes mountains, GEFS means are higher than CHIRPS means throughout the year.

The CHIRPS-GEFS downscaling procedure corrects for systematic errors in GEFS forecasts that vary spatially and temporally. To assess the efficacy of the CHIRPS-GEFS approach, we began by calculating the per-pixel difference between GEFS and CHIRPS, and CHIRPS-GEFS and CHIRPS for 15-day periods. These were calculated for each month, for in-season pixels, and then averaged across the year. We then looked at the histogram of the resulting differences (Fig. 1b), to identify the distribution of annual average errors in these two products. CHIRPS-GEFS errors are shown as gray bars and GEFS errors are overlaid as hollow red bars. A desirable pattern is more small errors (higher bars close to 0 mm) and fewer large magnitude errors (lower bars at larger precipitation values). As shown in Fig. 1b, the bias-correction procedure has this effect, and results in CHIRPS-GEFS having overall lower errors for global rainy seasons compared to GEFS. GEFS 15-day errors more commonly involve over prediction of observed amounts than under prediction, as shown by the higher proportion of positive versus negative moderate to large positive errors. Part of this is due to the lower limit of under prediction being zero precipitation, while over prediction can range from marginal precipitation amounts to very high amounts. As shown in Fig. 1b, the CHIRPS-GEFS bias correction particularly reduces GEFS forecast errors for moderate-to-high rainfall amounts, and it results in a global 15-day error distribution that has a higher proportion of small errors, e.g., errors within −10 mm to 10 mm of CHIRPS values (51% for CHIRPS-GEFS and 43% for GEFS). Errors in categories ranging from 10 mm to 40 mm occur less often in CHIRPS-GEFS, globally, with probabilities in those categories reduced by around 15 and 25 percent at 10 mm to 20 mm and 20 mm to 30 mm, respectively, and by around 30 percent to 40 percent for errors that are higher than 40 mm.

Next, we show performance of the 5-day and 15-day CHIRPS-GEFS precipitation forecasts by correlations and mean absolute errors for the historical record, compared to CHIRPS data for these periods. As described in Data Records, multiple outlets use forecast amounts for these periods. In the Usage Notes section, probability of detection scores for 15-day CHIRPS-GEFS in Africa are presented while describing an operational application of the CHIRPS-GEFS for seasonal monitoring. In that discussion we also examine the performance of 5-day forecasts during the 2020–2021 season in key regions of Kenya, Angola, Zambia, Zimbabwe, and Madagascar.

**Fig. 3:**
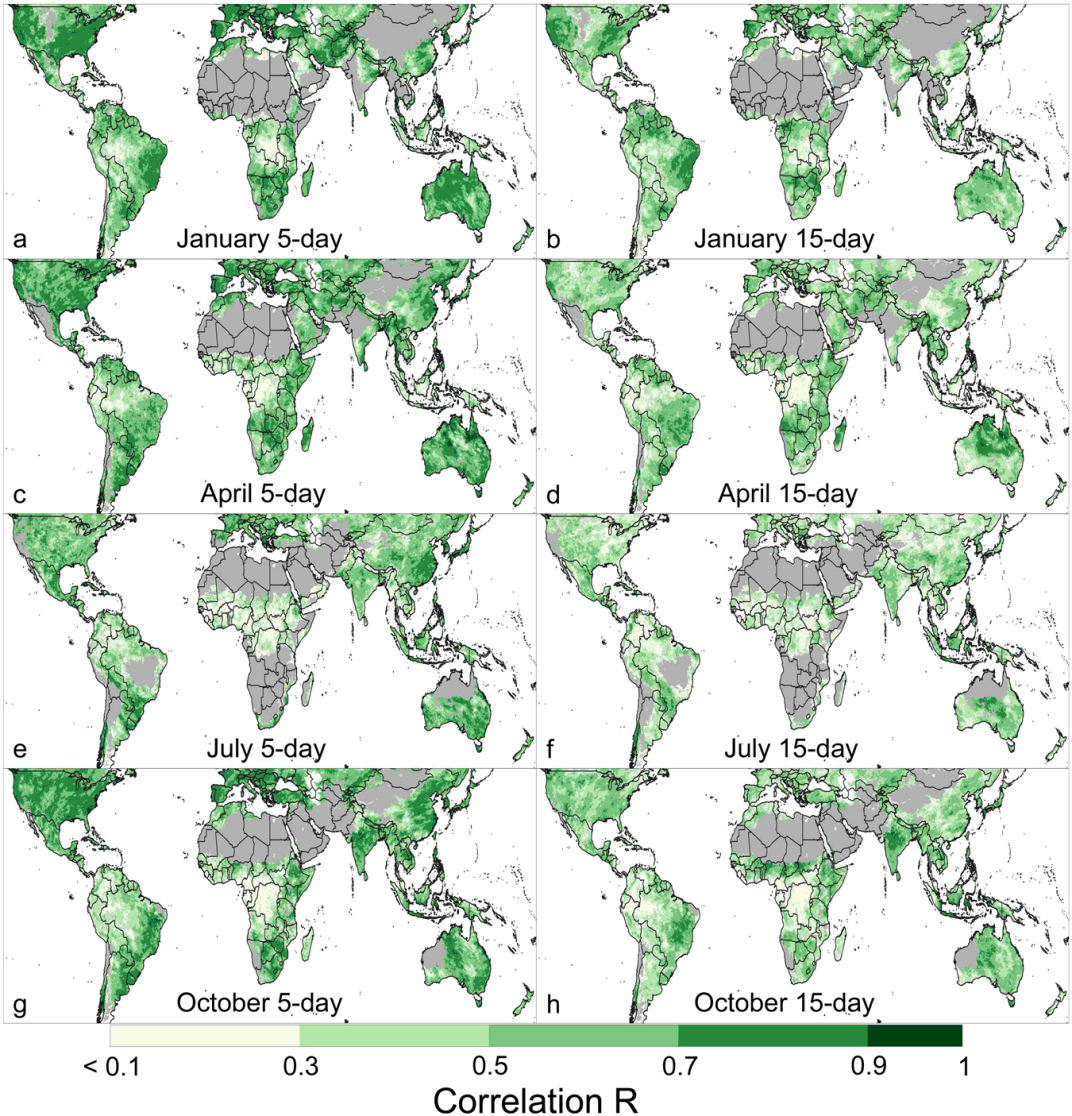
CHIRPS-GEFS 5-day and 15-day Pearson correlation coefficients, as compared to CHIRPS, for January, April, July, and October. (Validation data: CHIRPS 5-day and 15-day totals from the 1^st^, 6^th^, 11^th^, and 16^th^ of the month, for 2000 to 2019. Shown for in-season pixels, which are defined by monthly average CHIRPS > 10 mm.

Pearson correlation coefficients for 5-day and 15-day CHIRPS-GEFS, compared to CHIRPS (Fig. 3), indicate the ability of forecasts to predict deviations from average. It should be noted that correlations are nearly entirely driven by the information coming from the GEFS forecasts. The conversion to CHIRPS-GEFS adjusts the GEFS values to make them more “CHIRPS-like,” while also approximating the historical context of the GEFS forecast. Wet extremes forecasted by GEFS translate into wet extremes in CHIRPS-GEFS. Areas with very low correlations (R < 0.3) are where there should be low confidence in the forecasts, such as in parts of Central and West Africa and the Amazon region of South America. In many other areas, correlations for January, April, July, and October indicate moderate to good skill in forecast 5-day precipitation totals. In many of these areas, 15-day forecasts have lower but still identifiable skill. Some of the regions with primarily moderate (R 0.5 to 0.7) and high correlations (R > 0.7) are the United States, Western Europe, and Eastern Europe, southeastern South America, southern Central Asia, eastern China, parts of East and Southern Africa, and Australia. Globally, correlations are higher in January, April, and October than in July, which indicates generally higher forecast accuracy in those months. Exceptions are in eastern China, southern Brazil, eastern Mexico, northeastern Ethiopia, and central and southern Australia, where July correlations are not substantially lower. 15-day forecasts also have high correlations in some areas, including in the Western and Midwestern United States in January, in central and northern Australia in April, and in eastern Brazil in January and October.

In Africa, a region where CHIRPS data is actively used by the Famine Early Warning System Network (FEWS NET) and other organizations for seasonal monitoring and drought early warning, forecast correlations indicate moderate to good 5-day and 15-day forecast performance in areas of East Africa, Southern Africa, and western North Africa during rainy season months. Some of the highest 15-day correlations in Africa are during important rainy season months, for example, in northeastern Ethiopia in July and April, in Kenya in April, in Zimbabwe and southern Mozambique in January, and in the Sudanian zone of West Africa in October. Very low correlations indicate low forecast skill in the Sahel, coastal West Africa, and in Central Africa in the DRC, Republic of the Congo, and Gabon.

**Fig. 4:**
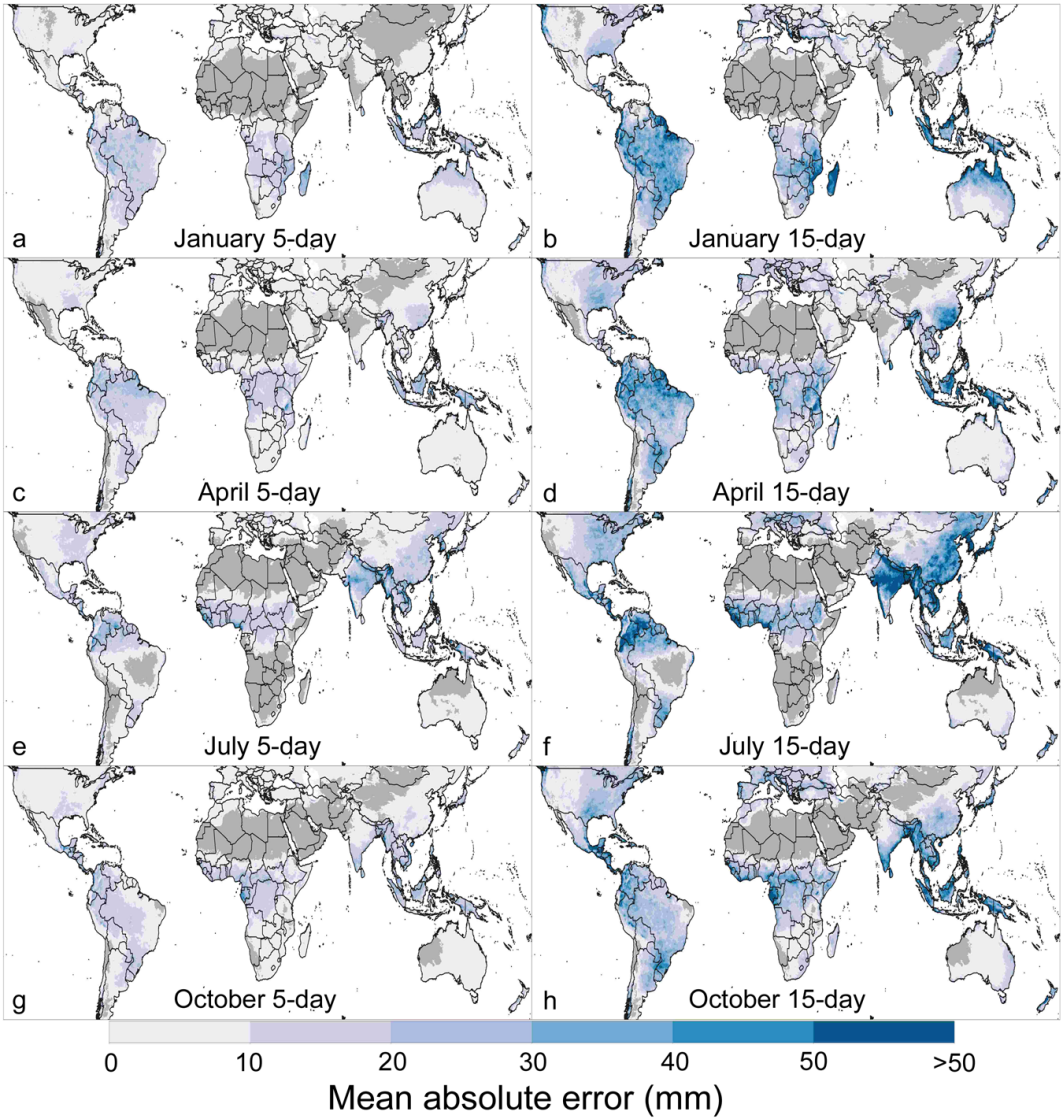
CHIRPS-GEFS 5-day and 15-day mean absolute errors, as compared to CHIRPS, for January, April, July, and October. Validation data: CHIRPS 5-day and 15-day totals from the 1^st^, 6^th^, 11^th^, and 16^th^ of the month, for 2000 to 2019. Shown for in-season pixels, which are defined by monthly average CHIRPS > 10 mm.

Mean absolute error of the bias-corrected GEFS forecasts highlight the areas where forecast amounts have historically been less reliable (Fig. 4). These indicate non-systematic errors associated with rains not materializing in the forecast location in the forecast period, which can be from GEFS model deficiencies and the inherent challenges of weather forecasting. Extreme precipitation events and warm season, deep moist convection-driven precipitation are notorious challenges for numerical weather prediction systems^48,49^, and CHIRPS-GEFS data are not immune to this problem. Remotely sensed data, including CHIRPS, also struggle with estimating extreme high rainfall amounts^13,50^, though since we are comparing CHIRPS-GEFS to CHIRPS, the main source of the large errors shown here would be the GEFS reforecast.

As shown in Fig. 4, the magnitude of errors follows climatology, with 5-day errors typically under 10 mm for drier rainy season months. In wetter months and locations errors are typically between 10 mm and 20 mm. With higher rainfall magnitude there is greater potential for larger errors. The 15-day forecast errors exhibit a similar spatial pattern to the 5-day errors, and error magnitudes correspond to the three-times larger accumulation interval as well as expected lower skill at longer lead time. Figure 4 shows especially large 15-day mean absolute errors in January near northern Mozambique and Madagascar, in July and October in parts of Central America, in April in central Kenya and southwestern Tanzania, in July in India’s Western Ghats Mountains and in the Himalayas, and in the Maritime Continent. In southeast China, while the 15-day correlations indicated decent skill at forecasting the sign of precipitation anomalies, large 15-day errors indicate the influence of poorly forecast large storms, which unbiasing cannot correct for. In the Amazon rainforest, many areas with low correlations also have high forecast errors, underscoring poor forecast performance there.

## Usage Notes

### CHIRPS-GEFS for operational hazards monitoring

One of many ways that CHIRPS-GEFS can support operational and custom forecast applications is as an extension to CHIRPS precipitation data. The compatibility of CHIRPS and CHIRPS-GEFS allows users to combine precipitation observations during the recent past and forecast amounts. By greatly reducing necessary bias correction, spatial downscaling, and temporal preprocessing steps, CHIRPS and CHIRPS-GEFS users can more easily use the products to assess the near-term risk of agrometeorological hazards like delayed growing season onset and prolonged mid-season dry spells and hydrologic hazards like flood risk.

Operational CHIRPS-GEFS applications that support drought early warning at FEWS NET include maps and time series graphics, updated every ~5 days, that show recent CHIRPS precipitation and an outlook using the next ~15 days (three pentads) of CHIRPS-GEFS forecasts. The Early Warning eXplorer, hosted by the United States Geological Survey (USGS) Earth Resources Observation and Science (EROS) Center, is an interactive viewer that shows these time series for administrative and cropping regions. CHC Early Estimates are maps and downloadable data, hosted by the UCSB Climate Hazards Center, which show recent and season-to-date precipitation performance linked with the forecast data (Table 1). In both these applications, incorporated forecast amounts are expected to be imperfect, and are plotted with clear distinction from recent data. Recent data comes from preliminary CHIRPS for recent weeks, based on satellite estimates and some station reports (source varies by region), and higher quality final CHIRPS for the previous month, which is blended with more station reports.

**Fig. 5:**
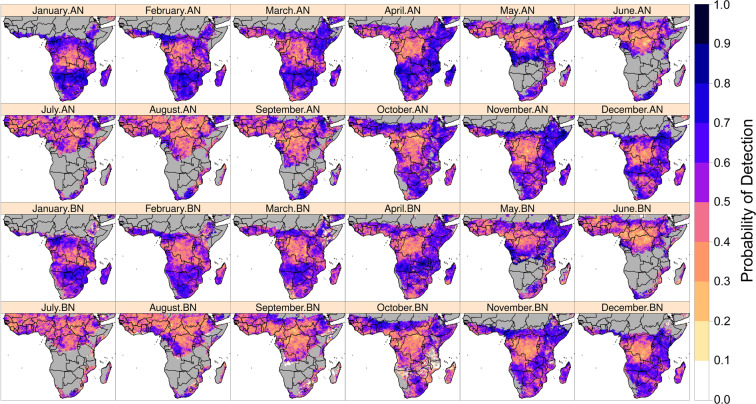
CHIRPS-GEFS 15-day probability of detection for above-normal (AN) and below-normal (BN) precipitation, as compared to CHIRPS, for January to December months in sub-Saharan Africa. Shown for in-season pixels, which are defined by monthly average CHIRPS > 10 mm.

A strength of the forecasts that these applications aim to capitalize on is if there are indications for substantial changes in areas with extreme or concerning precipitation conditions during recent weeks to months. Is an area that has been substantially drier than average since the start of their main rainfall season, or during key periods of rain fed crop development, forecasted to see wetter conditions? Or are deficits forecasted to intensify in the next two weeks, and further increase risks of severe crop moisture stress and low crop production outcomes? Are high precipitation amounts forecasted in an area that has been atypically very wet, which could increase the risk of flooding? Based on Fig. 5, which shows a 50% or higher chance of detecting above and below-normal 15-day precipitation (purple to black colors), with relatively high precision at 5-km resolution, the forecasts have enough skill to support these aims in many areas in sub-Saharan Africa. These scores are directly attributable to the GEFS forecast percentile, because CHIRPS-GEFS is derived from quantile-matching to GEFS. Seasons where the short-term outlooks in these applications can be regarded with some confidence include October-to-December Short Rains and March-to-May Long Rains in East Africa, February-to-May Belg rains in southwestern, central, and northeastern Ethiopia, Kirempt season in northeastern Ethiopia, and the October-to-May season in Southern Africa. As shown in Shukla *et al*.^51^, several of these regions are at higher risk of experiencing acute food insecurity.

### CHC Early Estimates and recent CHIRPS-GEFS performance

To help users understand the value of combining recent CHIRPS data with CHIRPS-GEFS, an example of the CHC Early Estimates and a focus on 2020–2021 forecasts is discussed here. CHC Early Estimates are accessible from the Recent and Seasonal Rainfall Monitors at the UCSB CHC website, from https://chc.ucsb.edu/monitoring. Users can view maps and download corresponding data showing precipitation totals, anomalies, standardized precipitation index (SPI) values, historical rank, and more for certain regions. These are updated every ~five days with the latest CHIRPS data. A “+Forecast” version provides a companion map that is a ~15-day outlook using the CHIRPS-GEFS forecast. The Regional and Seasonal Rainfall Monitors show cumulative precipitation during the most recent 1-, 2-, 6-, 12-, and 18-pentads (5 days to 3 months) and during region-specific agricultural rainfall seasons, respectively.

**Fig. 6:**
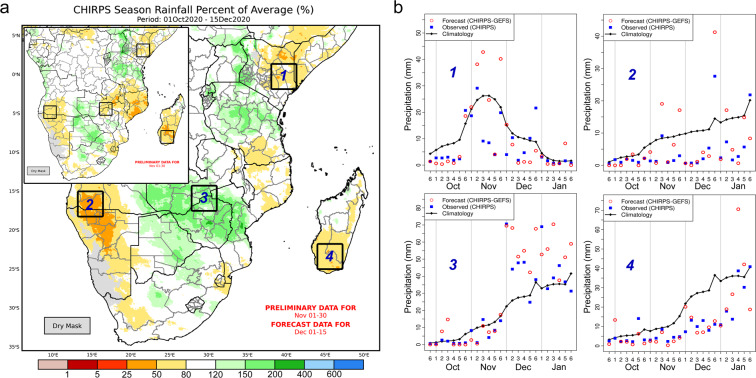
Operational CHIRPS-GEFS application example. (**a**) Operational applications of CHC Early Estimates for seasonal monitoring. Inset map shows observed rainfall for October 1st to November 30th, 2020, expressed as percent of average based on CHIRPS final and preliminary data. The larger map shows an Early Estimate extended 15 days later, based on the CHIRPS-GEFS forecast. (**b**) Performance of CHIRPS-GEFS pentad “Last” forecasts, during September 2020 to January 2021, for the areas outlined by boxes in panel a: Box 1, eastern Kenya; Box 2, southern Angola-northern Namibia; Box 3, southern Zambia-northern Zimbabwe; Box 4, southern Madagascar.

Figure 6 shows an example of CHC Early Estimates companion maps used to monitor seasonal precipitation conditions during late 2020 in Southern and Eastern Africa. In Fig. 6a, the inset map shows the percent of average precipitation for October 1^st^ to November 30^th^, 2020 based on CHIRPS final for October and CHIRPS preliminary for November. The large map shows how the October 1^st^ to December 15^th^ percent of average would look if the CHIRPS-GEFS 15-day forecast from December 1^st^ were to materialize. Four regions are outlined that food security analysts noted on December 2^nd^ when the map pair became available at the UCSB CHC Early Estimates Seasonal Rainfall Monitor. Several of these regions received attention during weekly and monthly FEWS NET seasonal hazards monitoring and food security outlooks due to the presence or anticipation of agricultural drought conditions associated with poor rainfall performance.

At the time of map production, the Short Rains season had been below average in eastern Kenya, and no substantial improvement was forecast (Fig. 6a Box 1). Early season rainfall deficits in southern Angola and northern Namibia were forecast to get substantially worse (Fig. 6a Box 2). A region of southern Zambia and northern Zimbabwe was forecast to see a notable wet change, with the first half of December bringing heavy rains that could eradicate early season deficits and result in above-average seasonal totals (Fig. 6a Box 3). Last, the Early Estimate maps indicated that forecast rainfall in southern Madagascar would lessen deficits somewhat, but that season-to-date rainfall would remain below-average (Fig. 6a Box 4).

Figure 6b shows how CHIRPS-GEFS “Last” (0-day lead) pentad forecasts compare to CHIRPS final pentad observations and climatology for these regions, from September 26^th^, 2020 to January 31^st^, 2021. Figure 6b shows that, in all four regions, several 5-day forecasts overestimated and underestimated pentad rainfall totals. During eastern Kenya’s Short Rains (Fig. 6b.1), there were two wet forecasts that did not materialize in November, one of which was completely wrong (below-average rainfall occurred). Despite these cases, the forecasts correctly predicted key features of the season, including a delayed start to the Short Rains (October in Fig. 6b.1), lengthy mid-season dry spells, and season-lasting below-average rainfall (Fig. 6b.2 and b.4), and a switch to above-average seasonal rainfall (December in Fig. 6b.3). These results highlight the use of CHIRPS-GEFS in seasonal monitoring and forecasting applications like these, and indicate that CHIRPS-GEFS could also be useful for applications focused on predicting rainfall season onset dates and dry spells. A high level of skepticism is appropriate in areas of the world where correlation maps, mean absolute error maps, and probability of detection scores indicate poor performance in 5-day and 15-day forecasts.

### Improved forecasts for agro-meteorological monitoring in Ethiopia

**Fig. 7:**
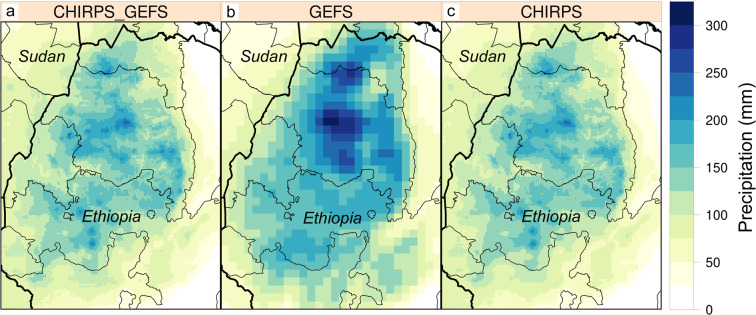
Enhanced spatial details in CHIRPS-GEFS in the Ethiopian Highlands region. (**a**) Mean forecast July 1st, 6th, 11th, and 16th 15-day precipitation total (mm) for CHIRPS-GEFS, during 2000 to 2019. (**b**) Same for GEFS reforecast. (**c**) CHIRPS v2.0 mean for same time periods.

The original motivation for CHIRPS-GEFS came from a NASA SERVIR-hosted workshop in Addis Ababa, Ethiopia. Discussions with Ethiopian meteorologists and agronomists highlighted their interest in a high-resolution CHIRPS-compatible weather forecast. This product would be suitable for guiding agricultural decisions, i.e., decisions about planting, fertilizer application, irrigation, etc. Figure 7 highlights how the CHIRPS-GEFS downscaling process injects detailed spatial information related to orographic rainfall enhancement. Using an example of mean 15-day forecasts over the Ethiopian Highlands region during July, one of the wettest months of the year, the CHIRPS-GEFS forecasts have much higher spatial definition than GEFS reforecasts (Fig. 7a versus b) and closely mimic the CHIRPS mean for the same time periods (Fig. 7c). Users can see further evaluation for Ethiopia at https://blog.chc.ucsb.edu/?p=443. This blog presents a station-based validation of GEFS and CHIRPS-GEFS dekads. CHIRPS-GEFS correlations are substantially better (0.68 versus 0.51) and root-mean-square errors substantially lower (37 mm versus 64 mm per dekad). CHIRPS-GEFS are actively used in dekadal agro-meteorological reports that are produced in collaboration with the Ethiopian National Meteorological Agency (https://chc.ucsb.edu/monitoring/ethiopia). These reports typically present the previous dekads’ CHIRPS observations and CHIRPS-GEFS forecasts. What is compelling in these results is how well the CHIRPS-GEFS captures the complex structure of precipitation. Furthermore, because these CHIRPS-GEFS forecasts are inter-operable with CHIRPS, it supports the rapid identification of mid-season deficits. The ability to reasonably capture orographic rainfall influences adds substantial value to GEFS.

### In-Development CHIRPS-GEFS Applications

Currently there are several operational grain forecasting efforts being developed based on CHIRPS products. Research has shown that CHIRPS precipitation estimates can be an accurate early season predictor of grain yields^52^ and that a key ingredient for improving grain predictions is to use sub-monthly rainfall totals (as opposed to monthly)^53^. We expect that combining CHIRPS with CHIRPS-GEFS forecasts in these grain forecasting systems can provide earlier indications for changing rainfall conditions and this could support reliable estimates made earlier in the season.

One promising area of novel early warning research where CHIRPS-GEFS may be particularly appropriate is the use of rainy season onset indicators as operational monitoring and forecasting tools. Shukla *et al*.^51^ found that CHIRPS-based start of season (SOS) indicators were strongly correlated with end of season remotely sensed Normalized Difference Vegetation Index (NDVI) data, a common measure of plant activity, in eastern and southern Africa. Likewise, Davenport *et al*. demonstrates that CHIRPS-based SOS could increase the accuracy of grain price forecasts in the same region. If CHIRPS-GEFS can be used simply to predict early or late seasonal onsets, the product could easily be used to reduce the latency of SOS-informed prediction models.

Finally, CHIRPS-GEFS is one of many products being ingested into the Defense Advanced Research Projects (DARPA) World Modelers (https://www.darpa.mil/program/world-modelers) Super Models as a Service (SuperMaaS) system (https://galois.com/project/supermaas/). This is a proto-type food security decision-support system that ingests data and models from a variety of services for real-time input into models for crop production, grain prices, conflict and other components of food security analysis. Both CHIRPS and CHIRPS-GEFS are currently registered in the SuperMaaS for both real-time viewing, analysis, and as inputs into related models.

## Data Availability

The Interactive Data Language (IDL) software was used to ingest the GEFS forecast data and the CHIRPS rainfall estimates, and to create the CHIRPS-GEFS output. IDL is a proprietary scripting language with a well-developed library of functions for interacting with large raster data sets, statistical analysis, and intuitive visualizations. IDL code used in producing CHIRPS-GEFS is available through the CHIRPS-GEFS^47^ data repository, at 10.15780/G2PH2M. Users can follow a link on that page to a directory that contains IDL pro scripts used in operational CHIRPS-GEFS production: (1) Retrieve GEFS GRIB forecast files from the NOAA NCEP GEFS website for days 1–10 from the 0.25 degree resolution output and days 11–16 from the 0.5 degree output; (2) Create 1-day and 16-day forecast precipitation accumulations from these files at 0.25 degree resolution; (3) For each pixel, identify the percentile rank of the current forecast compared to post-2000 reforecast and available “real time” GEFS for this time period, and then re-grid these to 0.05 degree to match CHIRPS resolution; (4) Produce a CHIRPS-unbiased version of the 16-day GEFS forecast by, at each pixel, sorting CHIRPS 16-day amounts for the same time period and identifying the CHIRPS amount matching the forecast percentile; and (5) Using these CHIRPS-GEFS 16-day forecast totals, produce 16 1-day totals based on the ratios of GEFS forecasts for each day versus the 16-day period.

## References

[1] Lorenz EN (1963). Deterministic nonperiodic flow. J. Atmospheric Sci..

[2] Hamill TM (2022). The Reanalysis for the Global Ensemble Forecast System, Version 12. Mon. Weather Rev..

[3] Zhou, X. *et al*. The Development of the NCEP Global Ensemble Forecast System Version 12. *Weather Forecast*. (2022).

[4] Funk C (2015). The climate hazards infrared precipitation with stations—a new environmental record for monitoring extremes. Sci. Data.

[5] Guan H (2022). GEFSv12 reforecast dataset for supporting subseasonal and hydrometeorological applications. Mon. Weather Rev..

[6] Nakalembe C (2021). A review of satellite-based global agricultural monitoring systems available for Africa. Glob. Food Secur..

[7] Funk C (2019). Recognizing the Famine Early Warning Systems Network: Over 30 Years of Drought Early Warning Science Advances and Partnerships Promoting Global Food Security. Bull. Am. Meteorol. Soc..

[8] Yang C, Yuan H, Su X (2020). Bias correction of ensemble precipitation forecasts in the improvement of summer streamflow prediction skill. J. Hydrol..

[9] Wood AW, Leung LR, Sridhar V, Lettenmaier D (2004). Hydrologic implications of dynamical and statistical approaches to downscaling climate model outputs. Clim. Change.

[10] Dinku T (2018). Validation of the CHIRPS satellite rainfall estimates over eastern Africa. Q. J. R. Meteorol. Soc..

[11] Beck HE (2017). Global-scale evaluation of 22 precipitation datasets using gauge observations and hydrological modeling. Hydrol. Earth Syst. Sci..

[12] Beck HE (2019). Daily evaluation of 26 precipitation datasets using Stage-IV gauge-radar data for the CONUS. Hydrol. Earth Syst. Sci..

[13] Harrison L, Funk C, Peterson P (2019). Identifying changing precipitation extremes in Sub-Saharan Africa with gauge and satellite products. Environ. Res. Lett..

[14] Katsanos D, Retalis A, Michaelides S (2016). Validation of a high-resolution precipitation database (CHIRPS) over Cyprus for a 30-year period. Atmospheric Res..

[15] Rivera JA, Marianetti G, Hinrichs S (2018). Validation of CHIRPS precipitation dataset along the Central Andes of Argentina. Atmospheric Res..

[16] Shrestha NK (2017). Evaluating the accuracy of Climate Hazard Group (CHG) satellite rainfall estimates for precipitation based drought monitoring in Koshi basin, Nepal. J. Hydrol. Reg. Stud..

[17] Duan Z, Liu J, Tuo Y, Chiogna G, Disse M (2016). Evaluation of eight high spatial resolution gridded precipitation products in Adige Basin (Italy) at multiple temporal and spatial scales. Sci. Total Environ..

[18] Duan Z (2019). Hydrological evaluation of open-access precipitation and air temperature datasets using SWAT in a poorly gauged basin in Ethiopia. J. Hydrol..

[19] Zambrano F, Wardlow B, Tadesse T, Lillo-Saavedra M, Lagos O (2017). Evaluating satellite-derived long-term historical precipitation datasets for drought monitoring in Chile. Atmospheric Res..

[20] Bai L, Shi C, Li L, Yang Y, Wu J (2018). Accuracy of CHIRPS Satellite-Rainfall Products over Mainland China. Remote Sens..

[21] Gao F (2018). Evaluation of CHIRPS and its application for drought monitoring over the Haihe River Basin, China. Nat. Hazards.

[22] Agutu NO (2017). Assessing multi-satellite remote sensing, reanalysis, and land surface models’ products in characterizing agricultural drought in East Africa. Remote Sens. Environ..

[23] Prakash S (2019). Performance assessment of CHIRPS, MSWEP, SM2RAIN-CCI, and TMPA precipitation products across India. J. Hydrol..

[24] Paredes Trejo FJ, Barbosa HA, Peñaloza-Murillo MA, Alejandra Moreno M, Farías A (2016). Intercomparison of improved satellite rainfall estimation with CHIRPS gridded product and rain gauge data over Venezuela. Atmósfera.

[25] Gummadi, S., Dinku, T., Shirsath, P. B. & Kadiyala, D. M. Spatial and Temporal Evaluation of Satellite Rainfall Estimates Over Vietnam. *Sci. Rep*. 10.21203/rs.3.rs-663644/v1 (In review).

[26] Wu W (2019). Performance evaluation of the CHIRPS precipitation dataset and its utility in drought monitoring over Yunnan Province, China. Geomat. Nat. Hazards Risk.

[27] Toté C (2015). Evaluation of Satellite Rainfall Estimates for Drought and Flood Monitoring in Mozambique. Remote Sens..

[28] Funk C (2015). A global satellite-assisted precipitation climatology, Earth Syst. Sci. Data.

[29] Peterson TC, Vose RS (1997). An overview of the Global Historical Climatology Network temperature database. Bull. Am. Meteorol. Soc..

[30] Zhou X (2017). Performance of the New NCEP Global Ensemble Forecast System in a Parallel Experiment. Weather Forecast..

[31] Zhou, X. *et al*. The Development of Next NCEP Global Ensemble Forecast System. In *Science and Technology Infusion Climate Bulletin* (NOAA’s National Weather Service, 2018).

[32] National Weather Service. *Public Information Statement 20-07*. https://www.weather.gov/media/notification/pns20-07gefs.pdf (2020).

[33] Hamill TM, Whitaker JS, Mullen SL (2006). Reforecasts: An Important Dataset for Improving Weather Predictions. Bull. Am. Meteorol. Soc..

[34] Hamill TM, Scheuerer M, Bates GT (2015). Analog Probabilistic Precipitation Forecasts Using GEFS Reforecasts and Climatology-Calibrated Precipitation Analyses. Mon. Weather Rev..

[35] Thielen J, Bartholmes J, Ramos M-H, de Roo A (2009). The European Flood Alert System – Part 1: Concept and development. Hydrol. Earth Syst. Sci..

[36] Cao Q, Shukla S, DeFlorio MJ, Ralph FM, Lettenmaier DP (2021). Evaluation of the Subseasonal Forecast Skill of Floods Associated with Atmospheric Rivers in Coastal Western U.S. Watersheds. J. Hydrometeorol..

[37] Voisin N, Pappenberger F, Lettenmaier DP, Buizza R, Schaake JC (2011). Application of a Medium-Range Global Hydrologic Probabilistic Forecast Scheme to the Ohio River Basin. Weather Forecast..

[38] Werner K, Brandon D, Clark M, Gangopadhyay S (2005). Incorporating Medium-Range Numerical Weather Model Output into the Ensemble Streamflow Prediction System of the National Weather Service. J. Hydrometeorol..

[39] Arsenault KR (2020). The NASA Hydrological Forecast System for Food and Water Security Applications. Bull. Am. Meteorol. Soc..

[40] Shukla S (2020). Improving early warning of drought-driven food insecurity in southern Africa using operational hydrological monitoring and forecasting products. Nat. Hazards Earth Syst. Sci..

[41] Gudmundsson L, Bremnes J, Haugen J, Skaugen TE (2012). Technical Note: Downscaling RCM precipitation to the station scale using quantile mapping–a comparison of methods. Hydrol Earth Syst Sci Discuss.

[42] Enayati M, Bozorg-Haddad O, Bazrafshan J, Hejabi S, Chu X (2021). Bias correction capabilities of quantile mapping methods for rainfall and temperature variables. J. Water Clim. Change.

[43] Jakob Themeßl M, Gobiet A, Leuprecht A (2011). Empirical-statistical downscaling and error correction of daily precipitation from regional climate models. Int. J. Climatol..

[44] Dettinger MD, Cayan DR, Meyer MK, Jeton AE (2004). Simulated hydrologic responses to climate variations and change in the Merced, Carson, and American River basins, Sierra Nevada, California, 1900–2099. Clim. Change.

[45] Hamill TM (2017). The US national blend of models for statistical postprocessing of probability of precipitation and deterministic precipitation amount. Mon. Weather Rev..

[46] NOAA Global Ensemble Forecast System (GEFS) Re-forecast. Accessed on October 10, 2020 from https://registry.opendata.aws/noaa-gefs-reforecast.

[47] Climate Hazards Center (2021). CHIRPS-GEFS.

[48] Herman GR, Schumacher RS (2016). Extreme Precipitation in Models: An Evaluation. Weather Forecast..

[49] Fritsch JM, Carbone RE (2004). Improving Quantitative Precipitation Forecasts in the Warm Season: A USWRP Research and Development Strategy. Bull. Am. Meteorol. Soc..

[50] Cavalcante RBL (2020). Evaluation of extreme rainfall indices from CHIRPS precipitation estimates over the Brazilian Amazonia. Atmospheric Res..

[51] Shukla S (2021). A slow rainy season onset is a reliable harbinger of drought in most food insecure regions in Sub-Saharan Africa. PLOS ONE.

[52] Davenport FM (2019). Using out-of-sample yield forecast experiments to evaluate which earth observation products best indicate end of season maize yields. Environ. Res. Lett..

[53] Lee, D. *et al*. Maize yield forecast using earth observation data and machine learning for Sub-Saharan Africa. *Glob. Food Secur*. **33**, 100643 (2022).

